# More Pi, anyone? Interplay between brassinosteroid signaling and the phosphate starvation response

**DOI:** 10.1093/plcell/koae080

**Published:** 2024-03-13

**Authors:** Vicky Howe

**Affiliations:** Assistant Features Editor, The Plant Cell, American Society of Plant Biologists; Department of Developmental Genetics, Heinrich-Heine University, 40225 Düsseldorf, Germany

Phosphorus is an essential plant macronutrient. However, the abundance of inorganic phosphate (Pi), the phosphorus form most readily absorbed by plants, is often depleted in intensively farmed soil. While the application of Pi-based fertilizer can remedy this, this short-term solution has detrimental long-term environmental impacts. Researchers are therefore interested in enhancing the innate mechanisms plants possess to cope with Pi deficiency. In search of potential genetic targets that could boost Pi absorption in rice (*Oryza sativa*), **Guoxia Zhang and coauthors** (Zhang et al. 2024) investigated the regulation of the Pi-starvation response (PSR) and identified an intersection between the PSR and brassinosteroid (BR) signaling pathway (see Fig. 1).

**Figure 1.**
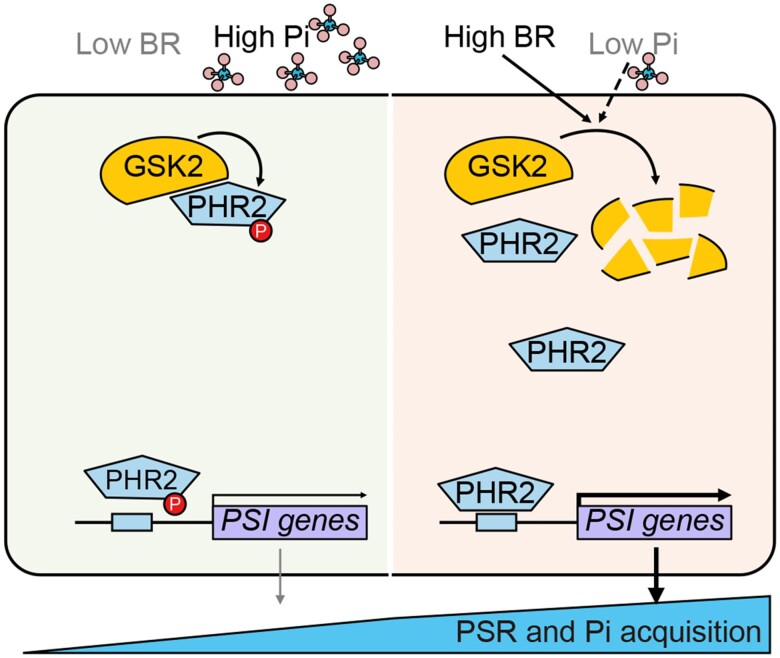
Schematic of the effect of BR and Pi levels on the PSR. Under low BR and high Pi (left), GSK2 phosphorylates OsPHR2. This hinders its DNA-binding activity toward Pi starvation–induced (PSI) genes and limits PSR. With high BR and low Pi levels (right), GSK2 is degraded, allowing transcriptional upregulation of PSI genes by OsPHR2 to increase PSR and Pi acquisition. Adapted from Zhang et al. (2024), Figure 6.

The BR signaling pathway has long been known to regulate plant physiology in response to developmental and environmental signals, including Pi availability. In Arabidopsis (*A. thaliana*), BR signaling mediates changes in root architecture in response to Pi starvation (Singh et al. 2014). In rice, Pi influences BR synthesis and signaling to modulate leaf inclination (Ruan et al. 2018; Guo et al. 2022). While these studies underscore the role of BRs in shaping plant architecture in response to Pi availability, the effect of the BR on PSR, as well as Pi utilization, is less understood.

In this study, Zhang and colleagues showed that, upon Pi starvation, rice mutants with overactive BR signaling had increased expression of classical Pi starvation–induced genes, suggesting that BR signaling amplifies PSR. This prompted the authors to examine PHOSPHATE STARVATION RESPONSE2 (OsPHR2), a major regulator of PSR. They found that the central inhibitor of BR signaling, GSK3/SHAGGY-LIKE KINASE 2 (GSK2), also downregulates PSR by phosphorylating OsPHR2 at a serine residue, S269. Thus, overactivation of BR signaling results in a stronger PSR, while BR and PSR can be simultaneously inhibited.

The group wanted to know how phosphorylation of OsPHR2 could lead to downregulation of PSR. They already knew that OsPHR2 binds to Pi starvation–induced genes via a conserved PHR1-binding sequence (P1BS) located in their promoters. As S269 is in a region required for this DNA-binding function, the authors wondered whether S269 phosphorylation hindered OsPHR2's ability to bind to the P1BS of its target genes. To test this, they created a mutant variant of OsPHR2 that mimicked phosphorylation at residue 269 and observed its P1BS-binding ability in vitro.

As predicted, the purified OsPHR2 phosphorylation-mimic did not bind well to small DNA probes consisting of the P1BS motifs from 2 target genes. Furthermore, plants overexpressing the OsPHR2 phosphorylation mimic had lower expression levels of other Pi starvation–induced genes. Together, this suggests that S269 phosphorylation reduces OsPHR2's ability to bind to and activate its transcriptional targets, thereby dampening PSR (see Fig. 1).

Next, Zhang and colleagues showed that rice plants with an overactive PSR (those overexpressing wild-type OsPHR2 or a variant unable to be phosphorylated at reside 269) displayed symptoms of Pi toxicity when grown with sufficient Pi. In contrast, plants overexpressing either OsPHR2 phosphorylation mimic, or both OsPHR2 and GSK2, appeared normal and had comparatively lower Pi contents. This confirmed the role of GSK2 in downregulating PSR and restricting Pi uptake. Moreover, subjecting GSK2 overexpressing plants to low Pi conditions led to a decrease in GSK2 protein levels, possibly by inducing proteasomal degradation, suggesting a feedback mechanism at the protein level by which Pi deprivation could upregulate PSR.

Finally, the group reasoned that, being upstream of GSK2, altering BR levels should have downstream effects on Pi levels. Indeed, mutants with impaired BR signaling had lower Pi levels, while mutants with enhanced BR signaling had higher Pi levels, demonstrating that upregulating BR signaling can boost PSR to increase Pi absorption. This study highlights the potential for synthetic application of BRs to increase Pi levels in crops while minimizing the use of Pi fertilizers. In addition, the OsPHR2 S269 phosphorylation site presents an intriguing gene-editing target to maximize Pi uptake in Pi-deficient conditions. What's more, this site appears to be conserved across angiosperms, so this could represent a promising target for multiple crop species. Ultimately, a detailed understanding of PSR will be necessary to breed more robust future crops. This study has contributed another piece to that puzzle.

## Dive Curated Terms

The following phenotypic, genotypic, and functional terms are of significance to the work described in this paper:

phosphorus CHEBI: CHEBI:28659
